# Seroprevalence of hepatitis B surface antigenemia and its effects on hematological parameters in pregnant women in Osogbo, Nigeria

**DOI:** 10.1186/1743-422X-9-317

**Published:** 2012-12-27

**Authors:** Olatunji M Kolawole, Abideen A Wahab, Daniel A Adekanle, Timothy Sibanda, Anthony I Okoh

**Affiliations:** 1 Applied and Environmental Microbiology Research Group (AEMREG), Department of Biochemistry and Microbiology, University of Fort Hare, Alice, South Africa; 2 Department of Biological Sciences, Osun State University, P.M.B 4494, Osogbo, Nigeria; 3 Department of Obstetrics and Gynaecology, LAUTECH Teaching Hospital, Osogbo, Nigeria

**Keywords:** Enzyme-linked immunosorbent assay, Hepatitis B surface antigen, Hematological parameters, Risk factors

## Abstract

**Background:**

The transmission of the hepatitis B virus (HBV) is parenteral, sexual and perinatal. Prevention of vertical transmission of HBV is extremely important because HBV infection in early life usually results in a chronic carrier State.

**Methods:**

A descriptive seroepidemiological study of hepatitis B virus and its effects on hematological parameters was investigated in pregnant women attending antenatal clinic of LAUTECH Teaching Hospital, Osogbo, Nigeria. 200 venous samples were subjected to full blood count and its sera were subjected to enzyme–linked immunosorbent assay for the detection of surface antigen of hepatitis B virus.

**Results:**

Prevalence rate of 16.5% was obtained for hepatitis B surface antigen in pregnant women. The highest HBsAg prevalence rate recorded was 23.3% for pregnant women between aged 30–34 years while the lowest recorded was zero percent for those aged greater than 40 years. RBC, WBC, neutrophil, hemoglobin lymphocyte and platelet counts have no significant effects on HBsAg positivity of pregnant women (p = 0.801). There was no significant difference in HBsAg positivity in relation to maternal age, gravidity, gestational age, family type, level of education and occupation (p = 0.073). Among the potential risk factors, there was significant difference in HBsAg positivity in the pregnant women in relation to their history of HBV vaccination (p = 0.039).

**Conclusions:**

We advocate universal free screening of pregnant women as the endemicity of HBV infections is thus being propagated.

## Introduction

Hepatitis B virus (HBV) is a double-stranded DNA virus belonging to Hepadnaviridae family. The incubation period is six weeks to six months [1]. Hepatitis B is a potentially life-threatening liver infection caused by hepatitis B virus. It is a major global health problem and the most serious type of viral hepatitis. It can cause chronic liver disease and put people at high risk of death from cirrhosis of the liver and liver cancer [2]. The primary method of transmission reflects the prevalence of chronic HBV infection in a given area. In low prevalence areas such as the continental United States and Western Europe, where less than 2% of the population is chronically infected, injection drug abuse and unprotected sex are the primary methods, although other factors may be important [3].

Transmission of hepatitis B virus results from exposure to infectious blood or body fluids containing blood. Possible forms of transmission include (but are not limited to) unprotected sexual contact, blood transfusions, re-use of contaminated needles and syringes, and vertical transmission from mother to child during childbirth [4].

In countries where HBV is highly endemic (hepatitis B surface antigen (HBsAg) prevalence rate of 8% or higher), most infections occur during infancy and early childhood. Infection occurs commonly in all age groups, although the high rate of chronic infection is primarily maintained by transmission during infancy and early childhood. Where endemicity is low (HBsAg prevalence rate of below 2%), infections occur in young adults, especially those belonging to known risk groups [5].

In areas with high HBV endemicity, perinatal is the main route of transmission. Perinatal transmission is common, especially when HBV infected mothers are also HBeAg positive . HBeAg – positive mothers are more than 70% while from HBsAg – positive, HBeAg negative mothers, it is less than 10% [6]. Edmund and colleagues supported this report that without intervention the risk of transmission from an HBeAg seropositive mother is 70-90% compare with the risk of about 10% from an HBeAg negative mother [7]. Nigeria is highly endemic and the most common circumstance that lead to HBV infection in this population have not been fully elucidated. It has been reported that the exposure rate to HBV (frequency of HBeAg and anti-HBs) ranged from 59% in children aged under 5 years to 72.5% in adults aged over 30 years, while the frequency of HBsAg alone was 40 and 10% respectively [8]. In most epidemiological studies age has always proved to be the most important factor. The age of acquiring infection is the major determinant of the incidence and prevalence rates [9].

Several vaccines has been developed for the prevention of hepatitis B virus infection. These rely on the use of one of the viral envelope proteins (hepatitis B surface antigen or HBsAg). The vaccine was originally prepared from plasma obtained from patients who had long-standing hepatitis B virus infection. However, currently these are more often made using recombinant DNA technology, though plasma-derive vaccines continue to be used; the two types of vaccines are equally effective and safe [10]. Following vaccination, hepatitis B surface antigen may be detected in serum for several days; this is known as vaccine antigenemia [11]. Vaccine is generally administered in a two, three or four dose schedules; and can be received by infants to adults. It provides protection for 85-90% of individuals and lasts for 23 years [12].

Various studies have shown that hematological parameters in pregnancy revealed variation from non-pregnant women [13-15]. Neutrophilia is a feature of pregnancy, while neutropenia is common among non-pregnant Africans [16]. Apart from the hematological changes brought about by hepatitis B infection in the mothers, both perinatal and maternal deaths are substantially increased in hepatitis B infection more especially in under priviledged populations and developing countries [2]. There appears to be a high incidence of low birth weight among infants born to mothers with acute infection during pregnancy [17]. About half of the babies born to mothers who have hepatitis B virus infection during pregnancy will show hepatitis B antigen in their blood and a proportion of them will develop hepatic lesions [18].

A yearly trend of HBsAg seropositivity in North-western Nigeria of 14.6% in 2004, 10.1% in 2005, 10.7% in 2006 and 11.4% in 2010 have been reported in blood donors [19]. A seroprevalence of 2.4% in the North-eastern, 2.19%, 51.9% and 4.3% in the South-western and South-southern Nigeria have been reported [20-23]. Recent studies in this locality in blood donors reported 13.5% HBsAg seropositivity [24]. However, to the best of our knowledge, studies on hepatitis B surface antigenemia in pregnant women have not been reported in this locality. Therefore, this study was aimed at finding the seroprevalence of hepatitis B surface antigenemia and its effects on hematological parameters in pregnant women attending antenatal clinic of LAUTECH Teaching Hospital, Osogbo, Nigeria.

## Materials and methods

### Study area

The research was carried out in Osogbo City. Osogbo is the capital of Osun state and is centrally situated in Osun State, Nigeria. Ladoke Akintola University Teaching Hospital was chosen as Sample Collection Centre.

### Study period

This study was carried out between December, 2010 and March, 2011.

### Sample size

The Fisher’s formula was employed which gives a total of 200 [25].

### Subject and samples

Two hundred pregnant women attending antenatal Clinic of LAUTECH Teaching Hospital, Osogbo in the South-western Nigeria were enrolled into this study after seeking their verbal consent. Ethical consideration approval with a reference number LTH/EC/2010/04/0118 was obtained from the hospital ethical review board. Serum samples were collected from pregnant women by venepuncture and stored frozen in aliquots at - 20°C until ready for use.

### Socio-demographic data

Subjects were verbally informed of the study and a questionnaire was administered to obtain socio-demographic information, such as maternal age, gestational age, gravidity, occupation, family type and level of education. Other information on risk factors to possible modes of transmission of HBV, such as history of previous blood transfusions, history of abortions, history of tattooing/tribal marks and history of HBV vaccination were obtained.

### Blood measurement

Hematological parameters considered in this study were; White blood cells, Red blood cells, Lymphocyte, Neutrophil, Hemoglobin and platelets. The process of complete blood count was determined by the use of an automated analyzer (Roche Sysmex Xe-2100).

### Assays

HBsAg was detected using third-generation enzyme linked immunosorbent assay (ELISA) kit (Biotech HBsAg device) in accordance with the manufacturer’s instructions. A repeat of ELISA test was performed on each positive HBsAg sample, in order to eliminate false positivity after carrying out a neutralization test for each positive test sample detected. Results were finally regarded as positive after a repeated positive ELISA test.

### Statistical analysis

Statistical software JMP version 9 (Generalized linear model) was used for all variables. Comparison were assessed using chi-square and t-test. A p-value of <0.05 was considered statistically significant in all statistical comparison.

## Results

Out of 200 sera from pregnant women tested for HBsAg, 33 (16.5%) were positive and 167 (83.5%) were negative. The highest prevalence of 14 (23.3%) was recorded in age group of 30–34 years while the lowest of 0 (0%) was recorded in age group greater than 40 years. This difference was not statistically significant (p = 0.171) (Table 1).

**Table 1:** Prevalence of HBsAg in relation with the age of pregnant women

**HBsAg**	**Age (years)**						**Total**
	15-19	20-24	25-29	30-34	35-39	>40	
Positive	1	4	13	14	1	0	33
	(14.3%)	(10.5%)	(16.9%)	(23.3%)	(6.3%)	(0%)	(16.5%)
Negative	6	34	64	46	15	2	167
	(85.7%)	(89.5%)	(83.1%)	(76.7%)	(93.7%)	(100%)	(83.5%)
**Total**	**7**	**38**	**77**	**60**	**16**	**2**	**200**

X^2^ = 4.7215, P-value = 0.4508 df = 5.

Also, the highest prevalence was obtained in multigravid 23 (20.7%) and the least prevalence 10 (11.2%) was obtained in primgravid. There was no significant difference (p = 0.073) (Table 2). However, 21 women at their third trimester had 17.4% prevalence rates while only one woman 20% at first trimester recorded a low prevalence rate (Table 3).

**Table 2:** Relationship between gravidity and hepatitis B virus transmission

**HBsAg**	**Gravidity**		**Total**
	**Primgravid**	**Multigravid**	
Positive	10	23	33
	(11.2%)	(20.7%)	(16.5%)
Negative	79	88	167
	(88.8%)	(79.3%)	(83.5%)
**Total**	**89**	**111**	**200**

X^2^ = 3.2516 P-value = 0.0714 df = 1.

**Table 3:** Relationship between gestational age and hepatitis B virus transmission

**HBsAg**	**Gestational age**			**Total**
	**1**^**st**^**trimester**	**2**^**nd**^**trimester**	**3**^**rd**^**trimester**	
Positive	1	11	21	33
	(20%)	(14.9%)	(17.4%)	(16.5%)
Negative	4	63	100	167
	(80%)	(85.1%)	(82.6%)	(83.5%)
**Total**	**5**	**74**	**121**	**200**

X^2^ = 0.2525 P-value = 0.881 df = 2.

In considering occupation, the highest prevalence rates was obtained in merchant women 14 (12.6%) and least prevalence was from housewives 0 (0%). Polygamous family presented highest positivity of HBsAg (22.2%) and (15.9%) recorded in monogamous family. Considering the level of education, the highest seropositivity of HBsAg 17 (17.2%) was obtained in women that had tertiary education while the lowest seropositivity of HBsAg 7 (25%) was recorded in women with primary education. There was no significant difference (p = 0.298) (Table 4).

**Table 4:** Relationship between socio-demographic characteristics of pregnant women with hepatitis B virus transmission

**HBsAg**	**P-value = 0.0913**		**X**^**2**^ **= 8.006 df = 4**			
	**Occupation**					
	**Civil Servant**	**Student**	**Housewife**	**Merchant**	**Medical Personnel**	**Total**
Positive	12	3	0	14	4	33
	(20.7%)	(16.7%)	(0%)	(12.6%)	(44.4%)	(16.5%)
Negative	46	15	4	97	5	167
	(79.3%)	(83.3%)	(100%)	(87.4%)	(55.6%)	(83.5%)
**Total**	**58**	**15**	**4**	**111**	**9**	**200**
	**P-value = 0.493**		**X**^**2**^ **= 0.4707 df = 1**			
	**Family Type**					
	**Monogamy**		**Polygamy**			**Total**
Positive	29		4			33
	(15.9%)		(22.2%)			(16.5%)
Negative	153		14			167
	(84.1%)		(77.8%)			(83.5%)
**Total**	**182**		**18**			**200**
	**P-value = 0.2956**		**X**^**2**^ **= 2.4374 df = 2**			
	**Education**					
	**Primary**		**Post-Primary**		**Tertiary**	**Total**
Positive	7		9		17	33
	(25%)		(12.3%)		(17.2%)	(16.5%)
Negative	21		64		82	167
	(75%)		(87.7%)		(82.8%)	(83.5%)
**Total**	**28**		**73**		**99**	**200**

The hematological indices (Table 5) of the pregnant women revealed that lymphocyte, RBC, WBC, neutrophil, hemoglobin and platelet counts were statistically non-significant with HBsAg positivity in the pregnant women (p = 0.51, 0.50, 0.49, 0.49, 0.51 and 0.48 respectively).

**Table 5:** Relationship of hematological indices of the pregnant women with hepatitis B virus transmission

**Parameter**	**HBsAg ± SEM**		
	**Positive**	**P-value**	**Negative**
Hemoglobin (g/dl)	10.08 ± 1.00	**0.51**	10.03 ± 1.28
Red blood cell (K/μι)	3.70 ± 0.47	0.50	3.72 ± 0.61
White blood cell (K/μι)	6.70 ± 1.63	0.49	6.78 ± 1.78
Platelet (K/μι)	182.52 ± 53.41	0.48	188.41 ± 55.49
Neutrophil (K/μι)	4.20 ±1.57	0.49	4.31 ± 1.40
Lymphocyte (K/μι)	1.86 ± 0.50	0.51	1.78 ± 1.52

Of those (71) with tattooing and tribal marks, 12.7% were HBsAg positive while there was none positive to HBsAg with history of previous blood transfusion. 5.4% were positive to HBsAg in those with history of abortion while 37.5% of those with history of HBV vaccination were positive to HBsAg in the pregnant women (p = 0.039).

## Discussion

Screening asymptomatic people is an important instrument in disease detection, prompt diagnosis and intervention, particularly at an early stage of the disease. This may improve the health outcome as well as better understanding of the transmission pattern of the disease [26]. In Asia and sub-Saharan Africa, HBV infection is endemic and thought to be the main etiological factor in over 75% of the chronic liver disease [24]. The results from this present study in Osogbo, Nigeria, revealed a seroprevalence rates of 16.5%, this lies within the established standard that West African countries have moderate to high hepatitis B endemicity as reported elsewhere [27,28]. High prevalence of 12% was reported among a similar study population in Taiwan and 10% in Hong Kong [29]. In Ilorin, prevalence rate of 5.7% was reported in mothers and 10% in their preschool age children [30]. In Benin city, a 2.19% maternal HBsAg seroprevalence have been reported [21].

The results indicated that the prevalence was high among 30–34 age-groups than others (Table 1). This correlates with the peak age of highest sexual activity in the society, hence supporting the role of sexual intercourse in the transmission of hepatitis B virus. The result also agrees with the report of Aganga [31] that in populations in which hepatitis B virus is relatively common; the majority of infections and peak prevalence of HBsAg as well as of specific antibody were in the age –group 25–29 and 30–34 years. There was no significant difference between age-groups as they relate to HBsAg prevalence (p = 0.171), therefore establishing the fact that HBV is common in all age-groups of life. However, the findings disagrees with the work of [32] in the same locality who reported highest HBsAg seroprevalence in blood donors of ages above 58 years.

The high prevalence of HBsAg in multigravids (20.7%) compared to primgravid (11.2%) might possibly be due to longer period of marriage and multiple deliveries in the locality (Table 2). This is in consonance with the report of Aganga [31] that women with longer period of marriage might have greater sexual activities and multiple deliveries, thereby exposing them to risk of HBV infection. There is a corresponding increase in HBsAg with an increase in the gestational age (Table 3). This might possibly be explained from the fact that pregnancy progresses with an attendant decrease in immunity of the expectant mother [33] a reason that might have given more chances to the virus to have undergone multiplication, a process that could result in the production of more HBsAg in the mother’s blood. There was no statistical significance of HBsAg positivity with gestatational age (p = 0.881). The high prevalence indicated among merchant women and those with tertiary education (Table 4), might possibly be explained by their high level of exposure and interaction with opposite sex. This could lead to having heterosexual partner, and this is one of the most important mode of transmission of HBV [34].

The risk factors identified in this study were tattooing / tribal marks, history of previous blood transfusion, history of abortion and history of HBV vaccination (Table 6). These findings are not unexpected since epidemiological studies have consistently demonstrated that transfusion of unscreened blood and blood products, and socio-cultural practices, such as circumcision and scarification, are important routes of transmission of HBV infection [35]. These socio-cultural practices are often done by the use of scientifically unsterilized devices. It is therefore plausible to suggest that engagement in these activities could have exposed the HBsAg-positive pregnant women to HBV infection initially. None of the pregnant women with history of previous blood transfusion were positive to HBsAg. This is not surprising as it is a national policy that all blood must be screened for HBsAg before transfusion in hospitals [32]. However, the significant association between HBsAg seropositivity in pregnant women in this study and history of previous HBV vaccination is in conformity with the findings by Alter and colleagues [36] who reported that no recognizable risk factor could be ascribed to 30% of cases of HBV infection in adults.

**Table 6:** Potential risk factors and prevalence of HBsAg among the pregnant women

**HBsAg**	**Risk factor**					**Total (%)**
	**Tattooing/tribal marks (%)**	**History of previous blood transfusion (%)**	**History of abortion (%)**	**History of HBV vaccination (%)**	**No risk factor (%)**	
Positive	9 (12.7)	0 (0)	2 (5.4)	3 (37.5)	19 (23.7)	33 (16.5)
Negative	62 (87.3)	4 (100)	35 (94.6)	5 (62.5)	61 (76.3)	167 (83.5)
**Total**	**71**	**4**	**37**	**8**	**80**	**200**

X^2^ = 10.7460 P-value = 0.0296 df = 4.

Out of the six hematological indices analyzed in this study, lymphocytes concentration were increased when compared with negative HBsAg pregnant women, though not statistically significant (p = 0.51). This could possibly be explained from the fact that the lymphocyte, which is one of the indices of white blood cells in combating diseases, is raised in the presence of viral infections [37]. However, this could be adduced to immunosuppressive status of women in pregnancy. The significant increased could also be aggravated by a dual infections in the pregnant women. HIV-HBV co-infected Nigerians have been reported to have lower CD4+ T-cell counts compared to HIV mono-infected individuals [38]. Several studies on HBV mono-infection support the idea that HBV leads to an overall increase in T-cell activation [39,40].

The major strength of this study is that to the best of our knowledge, this is the first report of seroprevalence of HBsAg and its effect on hematological indices of pregnant women in Osogbo and in the South-western part of Nigeria, a country known with high HBV endemicity. A limitation of this study is that we do not have data on opportunistic infections in the pregnant women prior to study entry.

In summary, in order to interrupt the cycle of transmission of HBV in Nigeria through perinatal route, these strategies would be recommended; free regular blood screening, public education, behavioural modification, passive immunoprophylaxis and active immunization.

## Conclusions

It is clear from this study that Nigeria is in the region with high prevalence of HBV. There are many unvaccinated women in childbearing age who are at risk of HBV infection. It is important to note that infection by HBV early in life underscores the potential of adding to the burden of viral hepatitis and its attending complication of hepatocellular carcinoma later in life.

## Competing interest

We wish to state that there are no actual or potential conflict of interest including any financial, personal or other relationships with other people or organizations that could inappropriately influence, or be perceived to influence, this work.

## Authors’ contributions

OMK and AAW participated in the design of the study and performed the investigation, analysis and interpretation of data. DAA participated in sample collection and administration of questionnaires. AIO participated in the design and coordination. OMK, AAW and TS drafted the manuscript. All authors read and approved the final manuscript.
